# Comparing noninvasive sampling techniques with standard cannula sampling method for ruminal microbial analysis

**DOI:** 10.3168/jdsc.2021-0094

**Published:** 2021-10-09

**Authors:** N. Indugu, M. Hennessy, V.S. Kaplan-Shabtai, C.F. de Assis Lage, S.E. Räisänen, A. Melgar, K. Nedelkov, X. Chen, J. Oh, B. Vecchiarelli, J.S. Bender, A.N. Hristov, D.W. Pitta

**Affiliations:** 1 Department of Clinical Studies, University of Pennsylvania, School of Veterinary Medicine, New Bolton Center, Kennett Square 19348; 2 Department of Animal Science, The Pennsylvania State University, University Park 16802

## Abstract

Rumen microbes play an important role in the conversion of indigestible plant material to energy and protein in dairy cows. Sampling for ruminal contents via cannula is considered the gold standard technique for microbial analysis, but the technique requires ruminally cannulated animals and specialized animal facilities. The purpose of this study was to determine whether other sampling methods and locations along the digestive tract may serve as noninvasive proxies to the cannula method for microbial analysis. Six ruminally cannulated lactating Holstein dairy cows were adapted to a standard total mixed ration for 2 wk and sampled during the third week. Sampling locations and methods included salivary content, rumination bolus (regurgitated digesta collected from the cow's mouth), feces, and rumen contents via stomach tube and cannula. Stomach tube and cannula samples differ in proportions of solid and liquid material and were therefore separated into whole (as collected), liquid, and solid fractions. Samples were collected at 0 (before feeding), 2, 4, 6, 8, and 12 h after feeding over 2 d. All samples were extracted for total genomic DNA and selected samples for metabolically active DNA (RNA), PCR-amplified for the V1-V2 region of the 16S rRNA bacterial gene, and analyzed for bacterial diversity using the QIIME2 pipeline followed by statistical analysis in R (https://www.R-project.org/). In DNA-based analysis, at the community level, saliva, rumination bolus, and fecal samples clustered in separate groups, whereas all fractions of stomach tube and cannula samples clustered together, indicating that microbial communities of stomach tube and cannula samples were homogeneous. Rumination bolus samples at 6, 8, and 12 h after feeding clustered with stomach tube and cannula samples, indicating that rumination bolus samples may be an alternative for cannula samples; however, time of sampling is critical for sampling of bolus digesta. Results of the RNA-based analysis of rumination bolus samples and solid samples from cannula and stomach tube at 0 and 6 h after feeding were similar. We concluded that the solid fraction of samples obtained via the stomach tube method may serve as a proxy for the solid fraction of whole ruminal contents obtained via cannula for DNA-based microbial investigations. Both rumination bolus and stomach tube solid samples may serve as proxies for cannula solid samples for RNA-based microbial analysis.

## Highlights

• Ruminal cannula is the gold standard for sampling rumen contents but is limited to few animals.
• Noninvasive methods are needed as proxy for cannula to enable sampling larger numbers of cows.
• Saliva, rumination bolus, tube-derived rumen samples, and feces were compared with cannula samples.
• Microbial community in the solid fraction of tube samples mirrored that of cannula samples.
• Rumination bolus may serve as a proxy for cannula samples under certain conditions.

## Graphical Abstract

**Figure:**
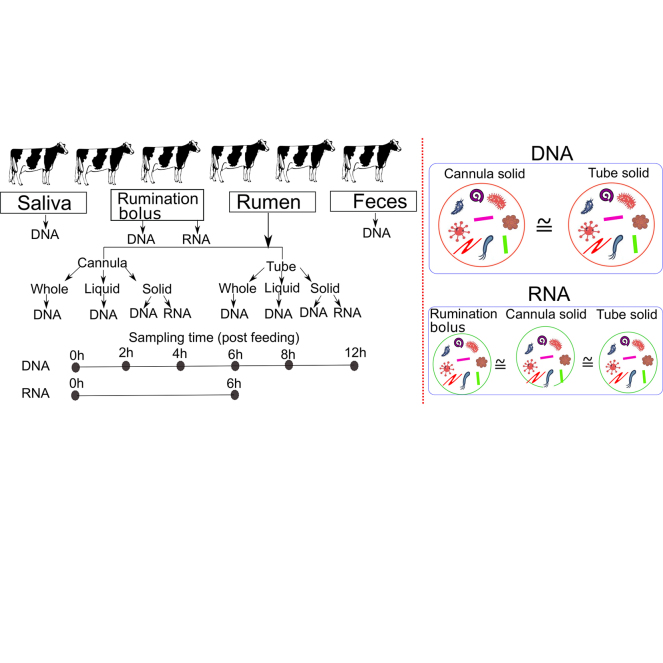
**Summary:** Rumen cannula is considered the gold standard sampling method for microbial analysis in dairy cows, but it requires cannulated animals. We investigated whether noninvasive sampling locations in the gastrointestinal tract could serve as a proxy for cannula samples. Stomach tube and cannula samples were separated into whole (as collected), liquid, and solid fractions. Samples were collected at 0, 2, 4, 6, 8, and 12 h after feeding over 2 d. All samples were extracted for total genomic DNA and a subset of samples for metabolically active DNA (RNA) followed by 16S rRNA amplicon-based bacterial diversity analysis. The solid fraction of tube-derived samples is a proxy for the solid fraction of cannula-derived samples based on DNA analysis. In RNA-based analysis, rumination bolus may serve as a proxy for the solid fraction of rumen cannula samples.

Microbial fermentation in the rumen is central to ruminant production by enabling the host to digest feed and provide nutrients required to make milk and meat (Sasson et al., 2017). Advances in next-generation sequencing technology have enabled the characterization of uncultured microbes from complex microbial ecosystems such as the rumen (Pitta et al., 2016a,b). Metagenomic and metatranscriptomic approaches in particular can provide insights into the functional aspects of microbes by determining changes in gene expression (Shi et al., 2014; Kamke et al., 2016). Although sampling methods including the use of cannulated animals, stomach tube, and rumenocentesis have been used to sample rumen digesta, studies comparing methods for microbial analysis are limited (Pitta et al., 2014a; Tapio et al., 2017). To exploit advances in genomic approaches in rumen microbial research, there is a critical need for noninvasive procedures that are easier to adopt and that allow for sampling larger numbers of animals in a limited time. Recent reports (Kittelmann et al., 2013; Tapio et al., 2017) have examined regurgitated ingesta (rumination bolus), oral swabs, rumen digesta sampled via cannula, and feces for bacterial, archaeal, fungal, and protozoal ecology in sheep and beef cattle. The authors reported that although the relative abundance of individual microbial populations varied with location, differences between animals and treatments appeared consistent, indicating that noninvasive methods may allow for large-scale screening of rumen samples for microbial composition. Furthermore, tube-derived samples have been found to be similar to cannulated samples but are composed mostly of the planktonic phase of rumen contents and may not represent the fiber fraction (Pitta et al., 2014b). Studies to compare ruminal microbiota at multiple time points before and after feeding in dairy cows using different techniques are needed to optimize sample collection times across different sampling techniques to effectively represent ruminal microbiota. We hypothesize that a core microbiota is present in the rumen of individual dairy cows and that a comparison of different sampling techniques at different times may enable us to determine a noninvasive method that can be used to sample larger numbers of animals. Therefore, the purpose of this study was to compare the microbial composition of samples retrieved by different techniques at different time points as well as different rumen fractions (whole/as collected, liquid, and solid) to identify an appropriate noninvasive proxy for cannula-derived rumen samples for microbial analysis.

The Pennsylvania State University Animal Care and Use Committee approved all animal-related procedures in this experiment (IACUC approval #48010). The details related to experimental design, library preparation, and bioinformatics methodology are described in de Assis Lage et al. (2020) and Kaplan-Shabtai et al. (2021). Briefly, 6 ruminally cannulated lactating Holstein cows fed a standard corn silage–based diet were enrolled in the experiment. Rumen samples were collected by 2 methods: rumen cannula (hereafter, **RC** samples) and stomach tube (hereafter, **ST** samples). Both RC and ST samples were immediately measured for pH, and a sample of the whole (as-collected) fraction was obtained before the remainder of the samples were separated into liquid and solid fractions by filtering through 4 layers of cheesecloth. For rumination bolus samples, each animal was observed for rumination behavior. Once the animal was observed to ruminate, portions of rumination bolus were removed by hand. Buccal saliva samples were collected using oral swabs as described in Kittelmann et al. (2015), and fecal samples were obtained directly from the rectum of the cows using a gloved hand. All samples were extracted for total genomic DNA and selected samples for metabolically active DNA (RNA), PCR-amplified for the V1-V2 region of the 16S rRNA bacterial gene, and analyzed for bacterial diversity using the QIIME2 pipeline followed by statistical analysis in R (https://www.R-project.org/).

For bacterial communities, approximately 25 million raw partial 16S rRNA sequences were obtained from 344 samples, with an average of 73,677 reads per sample and a range of 12,672 (min) to 132,716 (max) reads. Quality filtering and denoising of these raw reads produced a total of 41,413 amplicon sequence variants (ASV) for DNA and 10,706 ASV for RNA. Fewer than 100 reads per sample were observed in the negative control samples and they were eliminated from further analysis.

To identify an appropriate noninvasive proxy for cannula-derived rumen samples, we first investigated measures of diversity and evenness (α diversity), which did not reveal differences between different sample types. We next assessed differences in overall bacterial DNA community composition with a phylogeny-based weighted UniFrac metric (Figure 1) using principal coordinates analysis (**PCoA**) and permutational multivariate ANOVA (PERMANOVA) pairwise analysis for the 9 sample types. First, we found differences (*P* < 0.01) in the bacterial community composition between the solid and liquid ruminal fractions of RC samples, which agrees with Pitta et al. (2010). Second, we compared the bacterial community composition of whole, solid, and liquid ST samples. Interestingly, we found that ST solid was different (*P* = 0.001) from that of both ST liquid and whole samples but no differences were noted between liquid and whole fractions. This may be attributed to ST samples being predominantly composed of the planktonic phase, as described in Pitta et al. (2014a). Third, we compared solid, liquid, and whole RC fractions with the respective fractions of ST and found that bacterial communities of solid fraction in RC and ST were similar (*P* = 0.611), whereas liquid (*P* = 0.001) and whole (*P* = 0.001) fractions differed between RC and ST. We have previously discussed the comparison of bacterial communities and taxonomic composition between RC and ST for the liquid-only fraction (de Assis Lage et al., 2020) and the solid-only fraction (Kaplan-Shabtai et al., 2021). Collectively, these findings indicate that the solid fractions in both TS and CS samples mirror each other and, therefore, ST solid is a good proxy for RC solid. These findings agree with other reports that the solid fraction is resilient, whereas the liquid fraction of ruminal contents is vulnerable to external changes (Welkie et al., 2010; Lengowski et al., 2016). Finally, we compared these individual fractions with those of saliva, rumination bolus, and feces and found differences in bacterial community for saliva (saliva vs. RC whole, RC liquid, RC solid, ST whole, ST solid, and ST liquid; *P* < 0.01), rumination bolus (rumination bolus vs. RC whole, RC liquid, RC solid, ST whole, ST solid, and ST liquid; *P* < 0.01), and feces (feces vs. RC whole, RC liquid, RC solid, ST whole, ST solid, and ST liquid; *P* < 0.01) compared with rumen sample types. These findings are similar to those of several studies (Frey et al., 2010; Tapio et al., 2017; Noel et al., 2019) and clearly indicate that fecal and saliva bacterial communities are not suitable proxies for rumen bacterial communities.

**Figure 1:**
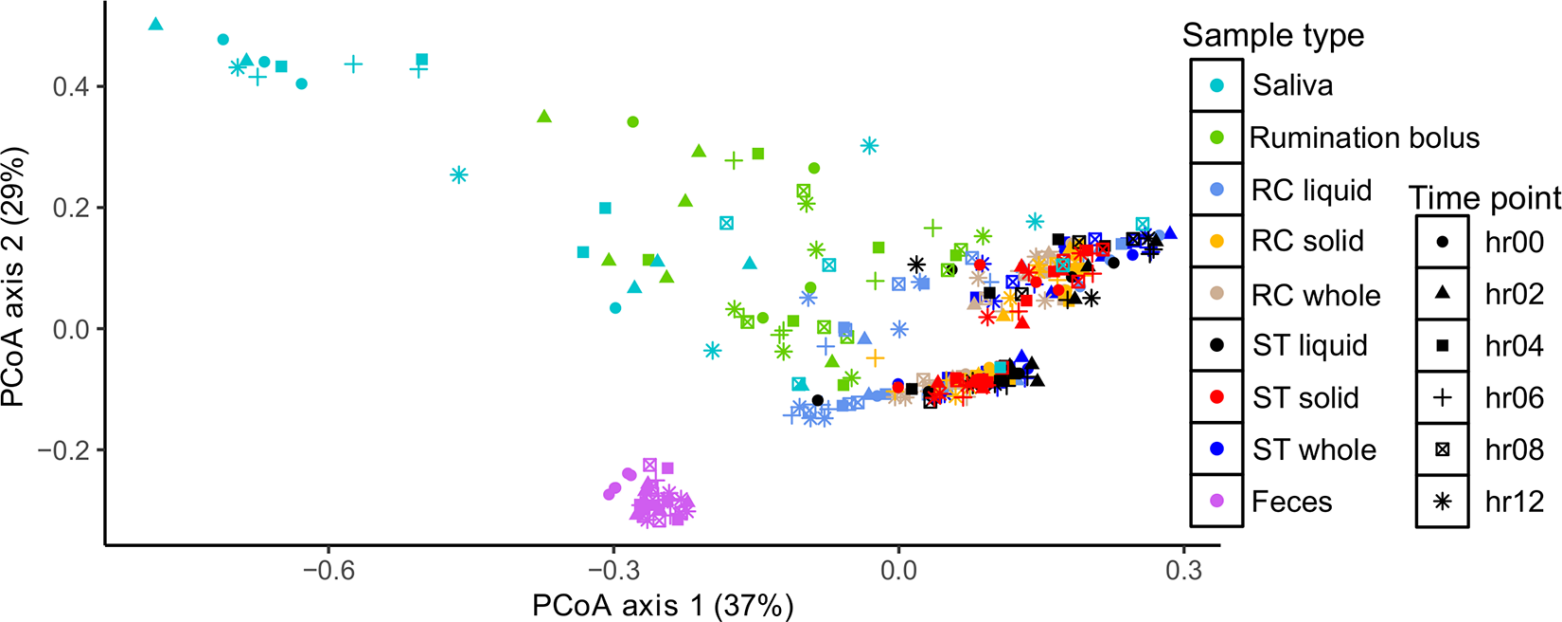
Principal coordinate analysis (PCoA) of samples based on weighted UniFrac dissimilarities of bacterial amplicon sequence variants in DNA samples collected from lactating Holstein dairy cows. Colors indicate sample types: saliva, rumination bolus, rumen cannula (RC) liquid, RC solid, RC whole, stomach tube (ST) liquid, ST solid, ST whole, and feces. Symbols indicate sampling time points (0, 2, 4, 6, 8, and 12 h post-feeding).

Next, we investigated the use of rumination bolus samples as a proxy for RC or ST solid samples. Ruminants regurgitate feed from the rumen to the mouth to allow reduction of particle size and microbial colonization (Gregorini et al., 2012). Ruminants spend 10 to 12 h/d ruminating on high-forage diets (de Boever et al., 1990), and this time may be lower in dairy cows fed mixed diets (Watt et al., 2015). We attempted to obtain samples when cows were ruminating as close as possible to the rumen sampling times. At the prefeeding (0 h) and 2 h postfeeding time points, the cows were not actively ruminating and, therefore, rumination bolus samples from 2 cows at 0 h were not obtained; however, we found that all cows were ruminating at 2 to 4 h after feeding. The DNA-based analysis (Figure 1) showed that only ST solid samples had close similarities with RC solid or RC whole samples. Although the community structure of rumination bolus samples was different from rumen samples, some rumination bolus samples (6, 8, and 12 h after feeding) were closely clustered with rumen samples based on PCoA (Figure 1). Therefore, we next examined whether the RNA-based bacterial community composition of rumination bolus samples could be a proxy for RC solid or ST solid RNA samples by analyzing RNA samples from rumination bolus, RC solid, and ST solid samples from 0 and 6 h and comparing them with corresponding DNA samples using PCoA (Figure 2) and pairwise PERMANOVA. At both time points, in DNA, rumination bolus community composition was different from RC solid and ST solid (*P* < 0.01), whereas in RNA, rumination bolus bacterial community composition was similar to both RC solid (*P* = 0.84) and ST solid (*P* = 0.94). There were no differences between RC solid and ST solid samples in either the DNA (*P* = 0.91) or RNA (*P* = 0.74) based approaches. Although bacterial communities in rumination bolus samples were significantly different from those of RC solid and ST solid at all time points (Figure 1), there was greater variation between rumination bolus, RC solid, and ST solid samples at 0, 2, and 4 h, whereas there was less variation between these sample types from 6 h onward.

**Figure 2:**
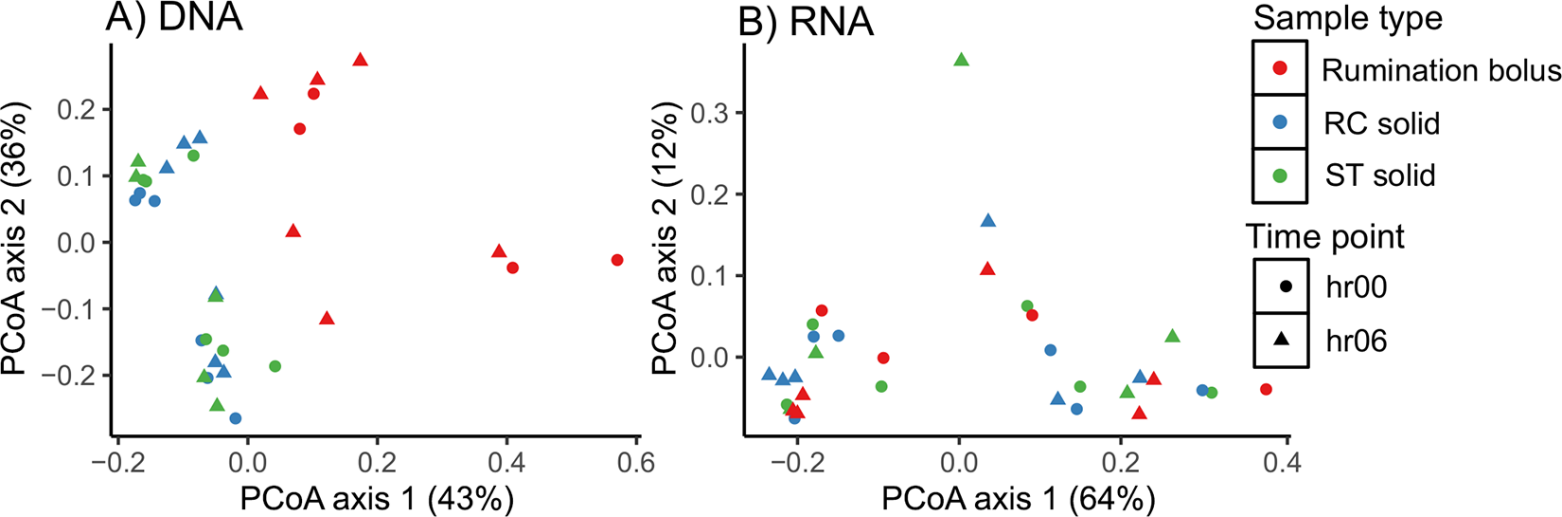
Principal coordinate analysis (PCoA) of samples based on weighted UniFrac dissimilarities of bacterial amplicon sequence variants in (A) DNA and (B) RNA samples collected from lactating Holstein dairy cows. Colors indicate sample types: rumination bolus, rumen cannula (RC) solid, and stomach tube (ST) solid. Symbols indicate sampling time points (0 and 6 h post-feeding).

In addition to compositional dissimilarity, we assessed differences in relative abundances of individual taxa associated with rumination bolus, RC solid, and ST solid samples in DNA and RNA-based approaches (Figure 3). *Firmicutes* was the dominant phylum in each sample type, followed by *Bacteroidetes*. For DNA, the third and fourth most prevalent phyla were *Actinobacteria* and *Proteobacteria* (rumination bolus and RC solid) and *Actinobacteria* and *Tenericutes* (ST solid). For RNA, *Proteobacteria* and *Fibrobacteres* were the third and fourth most prevalent phyla in each sample type. At the genus level, the most abundant bacterial genera are presented in Figure 3. In DNA, the most abundant genera on average in the rumination bolus samples were *Prevotella*, unclassified *Clostridiales*, unclassified *Lachnospiraceae*, *Solibacillus*, and *Butyrivibrio*, while in RC and ST solid samples, *Prevotella*, unclassified *Clostridiales*, unclassified *Lachnospiraceae*, *Butyrivibrio*, and *Ruminococcus* were most abundant. In RNA, *Prevotella*, *Ruminococcus*, unclassified *Clostridiales*, and unclassified *Lachnospiraceae* were the most abundant genera in rumination bolus, RC solid, and ST solid samples. The fifth most abundant genus was *Butyrivibrio* in RC solid and unclassified *Succinivibrionaceae* in rumination bolus and ST solid. Analysis of composition of microbiomes (ANCOM) analysis was used to identify differentially abundant genera between rumination bolus, RC solid, and ST solid in DNA and RNA. In DNA samples, 16 genera (*Rummeliibacillus*, *Solibacillus*, *Staphylococcus*, *Aerococcus*, *Carnobacterium*, *Enterococcus*, *Acinetobacter*, *Paenibacillus*, unclassified *Planococcaceae*, *Lysinibacillus*, *Paenisporosarcina*, *Desemzia*, *Lactobacillales*, *Leuconostoc*, unclassified *Lactobacillales*, and *Lactococcus*) were significantly different (Figure 3; ANCOM test) between rumination bolus, RC solid, and ST solid. These genera were present only in rumination bolus and were not present in RC solid or ST solid. In RNA, there were no significantly different genera between rumination bolus, RC solid, and ST solid, indicating the close similarity in composition between these sample types. The presence of several of these 15 bacterial genera in the rumen has not been reported before and because these genera were also detected in saliva samples, their presence in rumination bolus samples may be due to salivary contamination, which agrees with the findings of Kittelmann et al. (2013). We attempted to deplete bacterial genera that were common to saliva and rumination bolus but not present in RC solid and ST solid from rumination bolus samples to determine whether rumination bolus bacterial profiles were similar to RC solid and ST solid. However, despite intensive depletion steps, the differences between rumination bolus and RC solid or ST solid were not reduced (data not shown). The large variation between cows for the selected bacterial genera to be depleted made the depletion steps cumbersome. We conclude that rumination bolus samples obtained 6 h after feeding may show similarities in DNA-based analysis to DNA-based analysis of ST and RC solid samples, but the presence of background contaminants from the oral cavity may pose challenges in using rumination bolus samples as a proxy for rumen samples.

**Figure 3:**
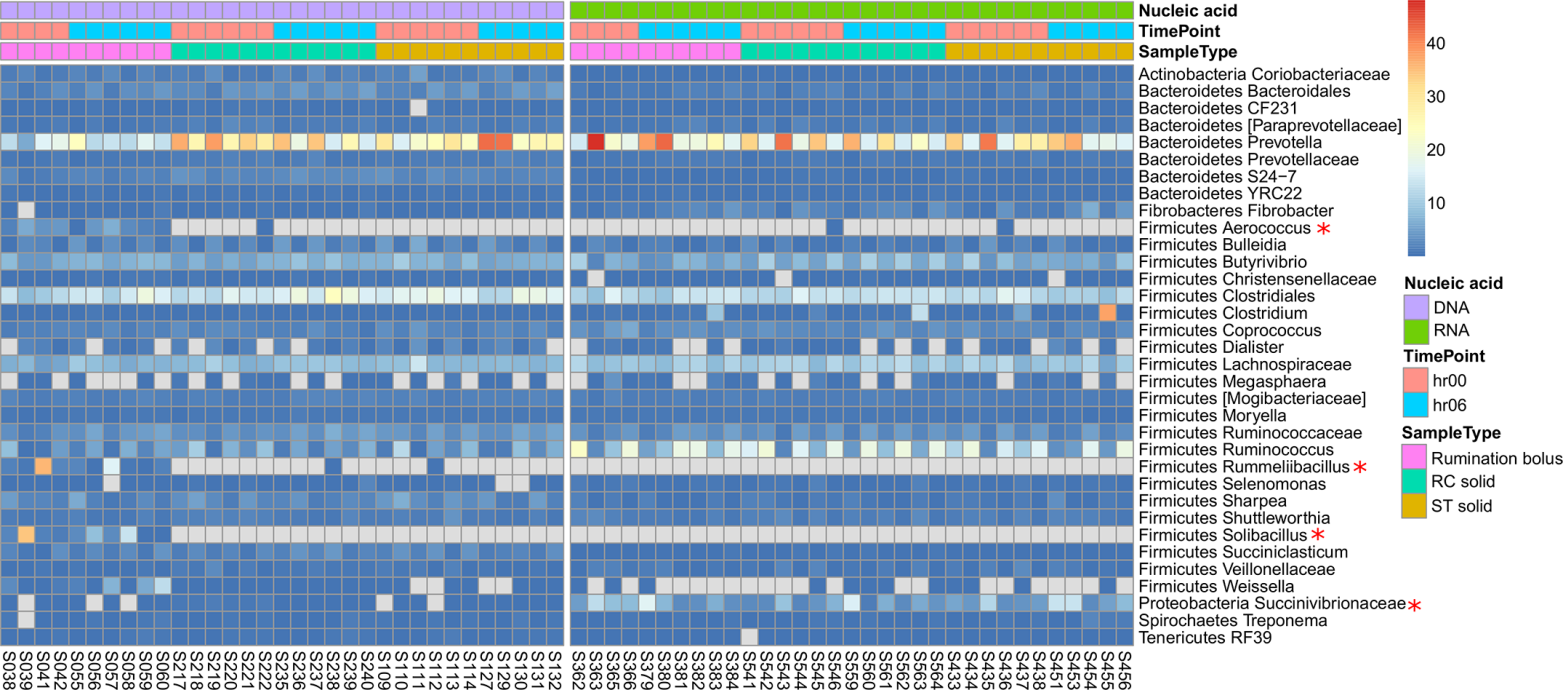
Heatmap showing the relative percentages of the most abundant bacterial genera in total (DNA) and metabolically active (RNA) samples of rumination bolus, rumen cannula (RC) solid, and stomach tube (ST) solid sample types at 0 and 6 h post-feeding. The asterisk (*) beside a genus name indicates the analysis of composition of microbiomes (ANCOM)-detected genera in total DNA.

Similar to DNA-based analysis, we detected no difference between RNA-based populations of RC solid and ST solid. In both RC and ST solid, a few bacterial populations including *Ruminococcus* and unclassified *Succinivibrionaceae* were more abundant in the metabolically active component than the DNA-based total component, confirming that analysis of RNA-based communities is more discriminatory than DNA-based analysis. Furthermore, the differences noted between rumination bolus and RC or ST solid in DNA-based analysis were not observed in RNA-based analysis, revealing that metabolically active populations are similar between rumination bolus, RC solid, and ST solid. Bacteria that appeared only in rumination bolus but not in RC solid and ST solid in DNA were not detected in the RNA component of the rumination bolus samples, indicating that they are dead or inactive cells that are present in the oral cavity or saliva. Collectively, these data indicate that targeting the metabolically active fraction in rumination bolus samples may be a good proxy for cannula samples.

To conclude, the filtered stomach tube fraction representing rumen solids is a better proxy for cannula-derived rumen solids compared with other fractions because both whole and liquid phases vary between time points. Rumination bolus samples can also be used as a proxy, but sampling for rumination bolus after 6 h post-feeding is recommended. Targeting metabolically active profiles provides a better representation of bacterial communities because DNA-based approaches incorporate background information from cells that are not necessarily viable. This study provides alternative sampling methods for rumen microbial analysis in dairy cows, thus allowing sampling of larger numbers of animals for microbial investigations.
